# Health services’ responses to transitioning adolescents to adult HIV care in South Africa

**DOI:** 10.4102/phcfm.v17i1.5060

**Published:** 2025-12-08

**Authors:** Charné Petinger, Talitha Crowley, Brian van Wyk

**Affiliations:** 1 School of Public Health, Faculty of Community and Health Sciences, University of the Western Cape, Cape Town, South Africa; 2 School of Nursing, Faculty of Community and Health Sciences, University of the Western Cape, Cape Town, South Africa

**Keywords:** adolescent health, HIV, transition to adult care, health system responses, peer support, healthcare worker perspectives, South Africa

## Abstract

**Background:**

Adolescents living with human immunodeficiency virus (HIV) aged 10–19 years account for 1.7 million globally, with 82% residing in sub-Saharan Africa. Older adolescents (15-19 years) assume greater responsibility for their own care, often leading to reduced adherence, lower retention, and poorer health outcomes. Understanding the role of healthcare workers (HCWs), key stakeholders in the HIV care continuum, is essential to strengthening transition practices and health system responses.

**Aim:**

To describe HCWs’ perspectives on transition practices for adolescents living with HIV in the Cape Town Metropole, South Africa.

**Setting:**

Six public primary health facilities in the Cape Town Metropole, South Africa.

**Methods:**

A descriptive qualitative design was used. Data were collected through in-depth, semi-structured interviews with 16 HCWs and analysed thematically.

**Results:**

Healthcare workers identified challenges to optimal transition (theme 1), including delayed disclosure, low adolescent readiness, and inconsistent transition processes. Health service responses (theme 2), such as youth clubs and provider-adolescent relationships, were supportive but unevenly applied. Gaps and recommendations (theme 3) included improving youth club management and ensuring system-wide support to enhance engagement and continuity of care.

**Conclusion:**

Successful transition to adult HIV care requires structural and psychosocial support mechanisms. Healthcare workers play a critical role and should be supported to consistently implement adolescent-friendly services during and post-transition.

**Contribution:**

This study offers system-level insights to inform policy, HCW training, and integrated models of care tailored to adolescents living with HIV in primary health settings in South Africa.

## Introduction

### Background

Globally, an estimated 39.9 million people were living with human immunodeficiency virus (HIV) in 2023, with adolescents representing a significant and particularly vulnerable subgroup within this population.^1^ Adolescents living with HIV, defined as those aged between 10 years and 19 years, account for approximately 1.7m individuals worldwide, with 82% residing in sub-Saharan Africa.^1,2^ In South Africa alone, an estimated 320 000 adolescents are living with HIV, placing the country among those with the highest population of adolescents living with HIV burden globally.^3,4^ Adolescence is a critical developmental phase marked by substantial biological, medical, psychological and social changes.^5^

As such, during this period, adolescents living with HIV are expected to transition from paediatric care to adult HIV care. The transition to adult HIV care is considered to be a vulnerable period wherein the health outcomes of adolescents are impacted.^6^ It is a purposeful, planned process addressing the medical, psychosocial and developmental needs of adolescents as they move towards the independent management of their health.^7,8,9^ For adolescents living with HIV, this shift is especially complex, given the ongoing challenges of disclosure, stigma, mental health challenges and structural barriers within health systems.^10,11,12^ A well-managed transition is essential to ensuring continuity of care, sustained antiretroviral therapy (ART) adherence and improved long-term outcomes. For older adolescents (15–19 years), this period often coincides with an increased expectation to independently manage their health, including their HIV care.^13^ However, the transition towards self-management is frequently accompanied by reduced adherence to ART and retention in care, which places adolescents living with HIV at a greater risk for virological failure and HIV-related morbidity.^14,15^ Consequently, it is evident that adolescents living with HIV in South Africa face disproportionately low rates of viral suppression and a higher risk of negative health outcomes.^6,16,17,18^

Healthcare workers (HCWs) play a central role throughout this process, as they serve as educators, care coordinators, providers of emotional support and gatekeepers to both paediatric and adult healthcare systems.^7,19^ Their ability to assess readiness, foster trusting relationships and provide adolescent-friendly care is key to a successful transition.^20,21^ When the transition is executed poorly, it results in interruptions in care and adverse health outcomes for the adolescent, such as virological failure and a higher risk of mortality.^15,22^ The above-mentioned risks occur when adolescents living with HIV are transitioned before they are ready or not adequately prepared or guided in the process of assuming self-responsibility for their care.^22,23^

There exists a need to examine transition practices from the perspectives of healthcare workers, as they are key stakeholders in ensuring the HIV care continuum and supporting adolescents through critical developmental milestones.^14^ Their insights are essential for understanding how the structure and responsiveness of the healthcare system either facilitate or hinder a successful transition, particularly in resource-constrained settings. Despite the increased recognition of the need for structured transition pathways, the absence of nationally standardised protocols continues to result in fragmented and inconsistent practices across healthcare facilities.^11,15,24^ In South Africa, a few studies have highlighted challenges such as poor retention after transitioning, healthcare worker hesitancy and the gaps in consistent adolescent-friendly service provision.^4,14,25,26^ However, it is imperative to explore transition challenges within the local context of public primary care service delivery in the Western Cape to inform contextually responsive interventions that align national policies with the complex and unique needs of adolescents living with HIV and the practical realities of healthcare service delivery.^14,25^ The aim of this study was to describe the perspectives of healthcare workers regarding transition practices for adolescents living with HIV in the Cape Town Metropole, South Africa.

## Research methods and design

### Study design

This study employed a qualitative descriptive design to describe first-hand experiences of HCWs and managers who provide care to adolescents living with HIV.^27,28^ This approach is valuable for describing individuals or events in their natural contexts, offering real-world practices and perspectives on the nature of the phenomenon.^29,30^

### Study setting

In 2020, the Cape Town Metropole had a population of approximately 637 353 adolescents between the ages of 10–19 years.^31^ It is characterised as a highly burdened area of people living with HIV, with 540 000 people on ART in 2022.^32^ This current study took place in all three levels (primary, secondary and tertiary) of healthcare services within the Cape Town Metropole. It is from six facilities, including three primary healthcare clinics and community health clinics (primary), two regional hospitals (secondary) and one hospital with all levels of care, referral facilities and specialised services (tertiary).^33^ These facilities provided both paediatric and adult HIV services to adolescents, three of which providing specific adolescent-centred care, such as youth clubs and scheduled visits after school.

### Study population and sampling strategy

Sixteen participants were purposefully sampled, based on their roles as healthcare workers or managers providing services to adolescents living with HIV within the public healthcare system in the Cape Town Metropole. The study sought to include healthcare workers who provide care to adolescents living with HIV and therefore included medical officers, nurses, counsellors and healthcare managers. The first few participants were identified by the authors based on whether they provide services in the Cape Town Metropole to adolescents living with HIV in public healthcare facilities, and subsequent participants were recruited *via* snowball sampling from the initial participants. All participants were contacted *via* email by the first author (C.P.) to recruit them into the study. Participants were recruited through direct invitations *via* email, based on an initial identification from the authors; further participants were recruited through snowball sampling. Maximum variation was sought across professional roles to capture a range of perspectives from both clinical and managerial viewpoints. Data saturation was determined after the 15th participant, drawing on Braun and Clarke’s notion of ‘theoretical sufficiency’.^34^ We established a sufficient sample size after the 15th interview, wherein thematic saturation was reached through the information-rich interviews.^35,36^ An additional 16th participant was interviewed to confirm this and thus it was validated sufficiently. The research team collectively determined that sufficient information power was obtained in the analysis.

### Data collection

We conducted in-depth individual, semi-structured interviews to collect data, using a semi-structured interview guide (see Online Appendix 1 for the interview guide). In total, 16 interviews were conducted. The interview guide was developed by the first author (C.P.) in consultation with the co-authors, as supervisors to the first author’s doctoral research and reviewed for clarity and relevance. The interview guide explored key areas with healthcare workers and managers, including the processes involved in transitioning adolescents to adult care at their facilities, their perspectives on what constitutes a successful transition, the challenges associated with providing paediatric and adolescent HIV care and the strategies they employed to address these challenges in service delivery for adolescents living with HIV. The first author conducted the interviews in person, with a second researcher, who were either of the co-authors (B.v.W. or T.C.) or a peer researcher (Y.M.). Co-authors (research supervisors) and an additional researcher occasionally attended interviews in a non-participatory role to enhance reflexivity and ensure consistency across data collection. Their role was to observe, provide feedback on interview technique and help monitor researcher positionality. Although they did not actively conduct the interviews, they are experienced qualitative researchers and were fully familiar with the study protocol and interview guide prior to data collection. Interviews lasted between 30 min and 60 min, were audio-recorded and were held at a place and time most convenient for the participants. Data collection took place over a 6-month period, from February 2024 to August 2024.

### Data analysis

Transcriptions of the interviews were uploaded to and were subject to analysis using the Atlas.ti software. This study employed reflexive thematic analysis, as developed by Braun and Clarke.^36^ This method assisted in comprehensively analysing data methodologically while maintaining the reflexive nature of the qualitative approach.^36^ Following these steps and developing codes and themes allowed the researchers to answer the research questions accurately and descriptively.^37^ C.P. and B.v.W. conducted initial coding of the data, and all the authors were involved in refining the coding framework.

### Rigour

Trustworthiness was maintained through the four concepts of Lincoln and Guba (1985), credibility, dependability, transferability and confirmability.^38,39,40^ Credibility is ensured through thick descriptions of the data, dependability is achieved through the consistency of the procedures utilised during data collection and the interview guide, rich descriptions of the participants’ experiences and contexts will inform transferability and confirmability is ensured through evaluating researcher bias.^29,39^ The first author kept a reflective diary, which was shared with the co-authors, throughout the data collection process to ensure that trustworthiness was maintained. In the interpretation of the study findings, we use verbatim quotes to ensure trustworthiness. Reflexive thematic analysis further strengthened trustworthiness, as reflexivity was embedded throughout the analytic process conducted by C.P. and B.v.W. This collaborative approach ensured that themes were iteratively refined through the team-based analysis process, thus strengthening trustworthiness and credibility of the findings.

### Ethical considerations

Ethical clearance to conduct this study was obtained from the University of the Western Cape Biomedical Science Research Ethics Committee (No. BM/23/6/5) and the Provincial Research Ethics Committee (WC_2023_048). Written and verbal informed consent was obtained from the participants of the study. Anonymity and confidentiality were maintained through the use of pseudonyms for participants and by redacting any identifying information.

## Results

### Participant characteristics

We included 16 participants in this study, all of whom were female healthcare workers within the Cape Town Metropole. We included five medical officers, four paediatric medical officers, two HIV counsellors, one HAST (HIV, AIDS [acquired immunodeficiency syndrome], sexually transmitted infections [STIs] and TB [tuberculosis]) manager, two clinical nurse practitioners and two who were employed as both clinical nurse practitioners and operational managers within their facility.^35,36^

### Themes

The emerging themes depict three overarching themes: (1) implementation challenges that hinder the optimal transition of adolescents living with HIV, (2) health services responses currently employed to support this process and (3) healthcare workers identified gaps and recommendations to strengthen the transition process and practices (Table 1).

**TABLE 1:** Coding framework.

Themes	Sub-themes	Code	Description of code
(1) Implementation challenges to an optimal transition	1.1. Expected challenges to adherence in adolescents living with HIV	Living with HIV is isolating	Adolescent experiences of HIV as explained by the HCW.
		Difficulty with self-management of chronic condition	Individual traits of the adolescent relating to managing their care, impacting retention in care, adherence and the transition process.
		Mental health issues	Participants struggle with mental illness, whereby readiness for the transition, as perceived by the HCW, is not achieved.
		Intellectual disability	Transitioning is delayed because of adolescents’ intellectual disabilities.
		Adolescent-specific factors impeding care	Unique adolescent-specific factors impeding their care and adherence that may be unique to adolescents.
	1.2. Barriers to effective transition	Partial disclosure of HIV status	Disclosure as a process that impacts adherence, engagement in care and eventually preparing for transitioning.
		Transition readiness	Aspects explicating transition readiness or the lack thereof.
		Held back in paediatric care	Adolescents being kept back in paediatric care by HCWs.
		Post-transition challenges	Experiences of adolescents’ performance and adherence after being transitioned to adult services.
(2) Health services responses	2.1. Peer and social support encouraged by HCWs	Peer mentors	Using peer mentors within adolescent support services assists in learning to navigate healthcare management and services.
		Peer relationships	HCW’s book adolescents living with HIV together as peer-to-peer psychosocial support helps mental health, adherence and acceptance of living with HIV.
		Recognising and facilitating familial support	The role of support, and the lack thereof, from the family of the adolescent.
	2.2. Healthcare provider support	Adherence support	If adherence support is given, then adolescents will be comfortable with their medication taking and clinic visits, but if this is not given, such as not engaging adolescents in deeper conversations about adherence, they may disengage from care.
		Healthcare provider support	Support measures put in place by healthcare workers to support adolescents living with HIV and their adherence such as home visits and phone calls, if adolescents are at-risk for disengagement from care.
		Healthcare provider relations	HCWs providing more adolescent-sensitive support and developing close bonds, which assists retention in care.
	2.3. Preparing the adolescent for transition	HCW providing knowledge on how the transition will occur	Providing adolescents with knowledge and support to physically and psychosocially manage the transition process.
	2.4. Teen clubs	Benefits of clubs	How youth or adherence clubs benefit adolescents and their health outcomes.
		Descriptions of clubs	Characteristics and procedures of the clubs.
(3) Recommendations for better transition	3.1. Implementing youth clubs as a tool for strengthening the transition	Acceptance of clubs	Adolescents’ acceptance of and attitudes towards being in youth clubs or groups.
		Developing and managing programmes	What goes into developing and managing clubs and programmes for adolescents living with HIV.
		Club criteria	Criteria adolescents living with HIV have to meet to be entered into youth clubs or groups.

HCW, healthcare worker; HIV, human immunodeficiency virus.

### Theme 1: Implementation challenges to an optimal transition

#### Sub-theme 1.1: Expected challenges to adherence in adolescents living with HIV

The participants describe significant barriers impacting adolescents living with HIV adherence and engagement in the transition process. At the core of these challenges was the experience of HIV as an isolating and stigmatising condition. Adolescents often reported feeling alone and misunderstood, especially if they did not know any other adolescents living with HIV. Participants explain:

‘So, teenagers would then come and say to me, no, they don’t know anybody else with HIV. They’re the only person.’ (Participant A, Female, Paediatric medical officer)

Therefore, it is evident from the experience of these healthcare providers that adolescents experience a sense of loneliness. Another barrier to remaining adherent relates to delayed disclosure of HIV status to the adolescents themselves. This may impact the adolescent’s acceptance of their condition, which directly affects their adherence to their treatment:

‘And if they haven’t been disclosed, they don’t have autonomy over, you know, their own sexual practices and knowledge and things like that. So, we are finding that disclosure to the child slash adolescent is something that needs to be worked on as well.’ (Participant C, Female, HAST Manager)

Delayed disclosure may be because of parental guilt for the transmission of HIV to their child. Participants explain that disclosure is often delayed because of mothers’ feelings of guilt. This is further strengthened, as participants explain that parental involvement during disclosure is necessary, and they should have support from the healthcare providers to assist with this process. When disclosure does occur, the parents’ fear may be validated, as it is found that adolescents may use treatment refusal as a form of retaliation. Participant J explains that ‘they think that if they don’t take [*their treatment*] that’s the method of punishing their parents’ (Female, Paediatric medical officer).

In addition to emotional and behavioural reactions, participants linked adolescents’ decreased adherence to broader psychosocial issues, such as mental health concerns, emotional immaturity and a lack of openness towards the healthcare provider. As Participant N observed:

‘She never told us why she doesn’t drink her pills. They usually don’t say, these young people.’ (Participant N, Female, HIV counsellor)

Participants felt that these individual traits combined with unstable living conditions, food insecurity and a lack of caregiver engagement further complicated adherence. Participant F articulates the emotional demands placed on healthcare workers to support adolescents living with HIV effectively:

‘I think there’s less emotional bandwidth for our kids and what they’re going through’. (Participant F, Female, Paediatric medical officer)

Many of these challenges are further exacerbated during the transition process, wherein services are often less flexible and less attuned to the developmental needs of adolescents. Participant K explains that adolescents may resist ongoing engagement in care, and when they transition to adult care, these services may not necessarily respond appropriately to their resistance. However, such adolescent-focused care is not available in current adult services in the public healthcare system.

#### Sub-theme 1.2: Barriers to effective transition

Beyond adherence, the transition itself was described as a major implementation challenge. Participants raised their concerns regarding whether adolescents were developmentally or emotionally ready for adult care, particularly those with psychological or intellectual disabilities. These individual-level factors were consistently referred to as reasons why adolescents living with HIV may disengage from care. Participant A supports this, noting that psychosocial often plays a role, not only in adherence, but in their experience of remaining in care throughout the transition process:

‘It’s generally always a psychosocial something.’ (Participant A, Female, Paediatric medical officer)

Participants drew attention to the stark contrast between paediatric and adult care environments. In paediatric care, *adolescents living with HIV* receive more personalised support, whereas in adult care, it is expected that patients are independent and able to manage their healthcare by themselves. These may be qualities many adolescents have not yet developed:

‘There’s a big difference even in age itself or personal life itself. Being a teenager and an adult. Yeah, it’s a huge, huge difference actually.’ (Participant A, Female, Paediatric medical officer)

Participants highlighted that this developmental gap is rarely accounted for in current transition practices. Oftentimes, adolescents are held back in paediatric services because of the healthcare workers’ hesitation and fear or disengagement. Some providers admitted to ‘holding on’ to their adolescents far beyond the appropriate age. This may also pose risks because of adolescents not being provided with the necessary skills to be independent and self-manage their illness. This also leads to adolescents not being ‘ready’ to transition:

‘But paediatricians are very bad at letting go of their babies. So, we have some teenagers who are still attending the baby clinic on a Friday morning.’ (Participant A, Female, Paediatric medical officer)

This hesitancy is often rooted in a perceived lack of readiness among adolescents living with HIV, particularly for adolescents with comorbidities or unstable psychosocial circumstances. Participants describe how adolescents often become accustomed to the support and flexibility of paediatric services, increasing the stark contrast of adult services:

‘So, we try to keep them because we found early on that if we worked with the adult club model, we lost teenagers who didn’t know what’s happening with them.’ (Participant G, Female, Medical officer)

‘Chance is that because they are already so used to easy, efficient, comfortable space I think they would default. They would. Yeah, it would be very difficult for them.’ (Participant H, Female, Clinical nurse practitioner/Operational manager)

This is furthered by the dependency adolescents have on their caregivers, which decreases their sense of responsibility. Participants are concerned about their patients’ ability to manage adult life and have a sense of agency and autonomy. This lack of autonomy was cited as a key reason as to why many adolescents living with HIV are deemed not ready to transition to adult care:

‘So that would be my biggest concern is how to get them to be functional adults that contribute to our society.’ (Participant E, Female, Medical officer)

Adolescents living with HIV may not have been provided the necessary skills and support to be able to speak for themselves, as Participant F mentions, ‘They don’t feel empowered to speak for themselves’ (Female, Paediatric medical officer). Consequently, delayed transitions often reflect healthcare workers’ concern about adolescents living with HIV’s emergence into adulthood, outside of their healthcare. Despite existing policies and age thresholds, healthcare workers frequently extended the paediatric care timeline as a result of the absence of adolescent-friendly transition support. This underscores a broader systems issue, which is that adolescents living with HIV are expected to transition prior to being fully prepared or supported to manage the complexities of adult HIV services. Tailored approaches are needed to address the unique psychosocial and developmental challenges adolescents face.

### Theme 2: Health services responses

This theme explores the healthcare services’ existing responses to supporting adolescents living with HIV during the transition. The following sub-themes will be discussed: peer support, healthcare provider support and relationships, social support and relationships, introducing the transition and preparing the adolescent and youth or teen clubs.

#### Sub-theme 2.1: Peer support and social support

Healthcare workers emphasised the importance of peer connections in mitigating isolation and supporting treatment engagement. Facilitating peer adolescents to feel less alone in their journey. Peer relationships allow adolescents to make friendships and also have a trusted source of their age to speak about HIV. Participant L ensures this through connecting participants, with their consent, *via* social media, ‘Yeah. We have a WhatsApp group. This is only for the youth who is active now’ (Female, Clinical nurse practitioner).

This also mitigates the feelings of isolation that may occur for adolescents living with HIV with the use of peer mentors – young people living with HIV – where already-transitioned adolescents can provide experienced support to their younger peers. This shows that adolescents can navigate their lives with the help of peers who have experienced similar situations. Furthermore, we find that enrolling adolescents living with HIV into youth clubs or groups assists with remaining engaged in care and facilitates the transition process. This further helped adolescents seek help more freely.

Participants also reflected on the role of caregivers and the broader family network in supporting adolescents living with HIV. Healthcare workers noted that when adolescents living with HIV have stronger familial involvement, they demonstrated greater adherence and stability. In some settings, efforts were made to integrate services for HIV-affected families to minimise logistical burdens and promote coordinated care:

‘Or, if the parent is also positive, then we have a club wherein we integrate them, so their dates are the same. So, we see the adult, doctor sees the child, but the date is combined so they don’t have to come on another day.’ (Participant O, Female, Clinical nurse practitioner/Operational manager)

Therefore, participants explain that strengthening familial relationships, as a healthcare provider, can prove helpful for adolescents living with HIV’s adherence. These efforts aimed to reinforce family cohesion and a reduction in stigma while promoting retention in care.

#### Sub-theme 2.2: Healthcare provider support and relationships

Many participants highlighted the role of building strong, trusting relationships with their patients. These provider–adolescent bonds were seen as instrumental in keeping adolescents living with HIV engaged in care:

‘The number one thing is creating relationships of trust.’ (Participant A, Female, Paediatric medical officer)

Establishing this trust was described as a slow, deliberate process, especially considering the developmental stage of adolescence. Participant B reinforced the importance of encouraging agency early on: ‘I do try to encourage speaking from an early age’ (Female, Medical officer). This is facilitated by ensuring that they develop relationships with the patients. This is a reciprocal relationship, as participants also feel attached to their patients, particularly as they witness improvement in the adolescent’s health. Participant M explains, ‘And one is proud of them, when you see how they progress in life’ (Participant M, Female, HIV Counsellor). In some cases, long-term care relationships began at birth, which fostered a deep continuity of care:

‘Yes, and the children open up to us, because as I said, you know these children, and one of them I even delivered.’ (Participant O, Female, Clinical nurse practitioner/Operational manager)

Counsellors who had worked with adolescents for an extended period were similarly able to form bonds that provided insight into the personal lives of adolescents living with HIV:

‘And the counsellors in the club have been there for a while. So, they’ve formed relationships with these adolescents. So, they know what’s going on in their lives as well.’ (Participant P, Female, Medical officer)

Despite these strong individual efforts, some participants were critical of the broader system’s lack of structured adolescent support, as Participant C mentions, ‘What extra support is there for adolescents? There isn’t any. I think people are just winging it.’ (Female, HAST Manager). This highlighted the reliance on individual initiatives rather than the systemic support for adolescent-centred care.

#### Sub-theme 2.3: Preparing the adolescent for transition

Several participants described the strategies they employed to prepare adolescents for the transition. These included a gradual orientation regarding the responsibilities of managing their treatment and normalising communication between the provider and adolescent. For example, participants ensure that adolescents remain in care through carefully paying attention to adolescents living with HIV by ‘recalling and hunting and finding you and keeping you in care’ (Participant B, Female, Medical officer). Participants noted that early preparation and open conversations about future care expectations were essential in building confidence and readiness.

Thus, participants expressed the importance of introducing autonomy and life skills as early as possible, even informally such as self-expression during consultations. This preparation was described as critical in helping adolescents living with HIV build the confidence needed for adult services, where support is less structured.

#### Sub-theme 2.4: Teen clubs

Youth clubs were widely cited as one of the most effective mechanisms for supporting adolescents throughout their HIV journey. These clubs provided a structured, adolescent-friendly environment where adolescents living with HIV could receive care, engage in peer interactions and participate in educational and psychosocial activities. Healthcare workers observed that adolescents who were actively involved in youth clubs were more likely to remain in care, remain adherent and navigate the transition process more effectively. Participants explain that the personal growth being developed through these groups also helps with adherence. Participant L furthers this, ‘so they motivate each other’ (Female, Clinical nurse practitioner).

Despite its benefits, youth clubs are not universally accessible and are often limited to adolescents who meet specific clinical criteria. Some participants also raised concerns about the sustainability of club models because of staffing and resource constraints.

### Theme 3: Recommendations for better transition

As discussed above, healthcare workers recognised youth clubs as a valuable intervention to support adolescents living with HIV during the transition. However, participants also identified several gaps in implementing and maintaining these programmes. This is described in the sub-theme, *gaps and recommendations in implementing youth clubs*¸ wherein adolescents’ acceptance of clubs, the operational challenges in developing and managing clubs and restrictive enrolment criteria that may exclude vulnerable adolescents.

#### Sub-theme 3.1: Implementing youth clubs as a tool for strengthening the transition

While many adolescents were described as benefitting from peer interactions and structured support in youth clubs, participants noted variability in how these were received. Some adolescents embraced the opportunity to engage with others, gain integral HIV knowledge and receive peer motivation. Participant P explains:

‘Yes, I do think so. I think in general, the adolescents, they also, I think, respond to a peer support system quite well.’ (Participant P, Female, Medical officer)

Others, however, demonstrated reluctance or disinterest. Participant D shared that despite attempts to introduce youth groups, adolescent participation was limited by a lack of motivation to engage. This highlights a broader challenge in fostering adolescent buy-in, which is essential in sustaining peer-led models.

Our findings indicate the benefits of managing youth clubs and enrolling adolescents in them to improve their adherence and growth, to assist with transitioning to adult care. However, youth clubs were reported to be difficult to implement in all facilities because of various implementation factors, including higher-level managerial buy-in, as explained by Participant B:

‘So, buy-in from the stakeholders and implementing actors and um, I think also, um, management and leadership is very important because if your people believe you and trust the person that is saying, let’s do it this way. Um, it’s very key to like policy implementation … So, how you do it.’ (Participant B, Female, Medical officer)

Supplementing traditional clinical care with an additional peer network may facilitate growth for the adolescents. Further, Participant H discusses this increased adherence as a result of being in clubs, because ‘they are more disciplined’ (Female, Clinical nurse practitioner/Operational manager). This may be because of an increased opportunity for improving HIV knowledge. This also assists with the transition process, whereby adolescents may remain together, even after being transferred to adult services. The groups also assist in readiness for adulthood, outside of their clinical care, through personal and professional growth.

Adolescents living with HIV receive additional support through these youth clubs, and in some cases, these spaces are also supported by external partners:

‘Actually, when you say it out loud, that’s what we are doing. Yeah. And the other difference between the adolescent club and the adult club is that there is something called the overseeing partners. So, the orphan vulnerable children are community partners. They are also NGOs. And at least they can also support the adolescent clubs. Say if you want to have an event, like a youth day event, there would be that organisation would be recruited to maybe help with refreshments or a health talk or something. And they have their own Kids Alive programme, which they also try to enrol the youth club patients in.’ (Participant P, Female, Medical officer)

The role of external partners, including community-based organisations and NGOs, provided support that added value to the implementation of youth clubs. However, the participants noted that the reliance on external actors could make the programmes vulnerable to disruption or termination when it is not adopted into healthcare services or are reliant on external financial support.

Another key barrier identified by participants was the use of restrictive criteria for enrolment into youth clubs. In some facilities, participants explain that only adolescents who were virally suppressed or stable on treatment were eligible for youth clubs. Participants further raised their concerns that the club criteria excluded adolescents who were most vulnerable and in need of psychosocial support and structured adherence interventions. Limiting access to only stable patients may inadvertently reinforce disparities in care.

Therefore, a peer support system combined with clinical care is beneficial to ensuring that adolescents remain in care and adherent; however, in order to do so, buy-in is necessary from the health system as well as the adolescents themselves. Despite the participants’ increased efforts to implement mitigating strategies to improve care for adolescents living with HIV who are at different stages of the transition process, there are barriers to overcome at both the psychosocial and health system levels.

## Discussion

The findings of this study illustrated that the transition to adult HIV care for adolescents living with HIV is not a single event, but a multifactorial process. Our participants, who were healthcare workers providing care to adolescents living with HIV, highlighted the challenges faced by adolescents pre-, during and post-transitioning to adult HIV care. These challenges provide important insights into the health service responses, implementation challenges and healthcare workers’ recommendations.

Healthcare workers described several existing systemic and service delivery challenges that continue to undermine the effective transition to adult HIV services. This subsequently includes the adolescent-specific factors impacting adherence. Participants described how adolescents living with HIV often lacked self-management skills, HIV literacy and mental health support. These barriers are also emphasised in existing literature within this context.^8,10,21^ Adolescent-friendly service models should integrate flexible clinic hours and adherence clubs, which can significantly improve access to ART and retention in care.^11,20^ However, these are not consistently implemented across facilities, as seen in this study.

During the transition process, participants reported that adolescents were sometimes held back in paediatric care because of the subjective assessments of readiness. These echo concerns raised in previous studies on how the lack of structured, evidence-based transition protocols impacts the way transition is implemented, as a process.^10^ Early planning, youth-friendly providers and consistent follow-up post-transition can be paramount in ensuring a successful transition.^8^ Albeit, adult HIV care often lacks the resources and support structures to meet adolescents’ unique psychosocial needs.^15,41,42^ The findings of this study confirmed this disconnect, with adult clinics frequently being unprepared to provide adolescent-friendly care. This is particularly strengthened by healthcare workers’ hesitancy to transition adolescents living with HIV, as they anticipate disengagement from care. Structural factors, such as the absence of adolescent-dedicated spaces and under-resourced clinics, can diminish trust and play a role in the disengagement from care for adolescents living with HIV.^8,11,43,44^

After the transition, adolescents living with HIV often experience poor adherence and disengagement in care, particularly when they are unsupported.^13^ Our findings strengthen evidence showing that adolescents who transition without adequate psychosocial and structural support are at a greater risk for interruption in ART care and loss to follow-up.^10,45^ Positive relationships with providers, the presence of peer mentors and ongoing psychosocial support post-transition were seen as protective factors by healthcare workers.^33^ These findings complement those from an earlier phase of the first author’s doctoral research, which used a quantitative survey to map transition readiness in the Cape Town Metropole, South Africa.^33^ Notably, the participants called for the transition to be reframed as a process that is embedded within routine service delivery, rather than marking it as a shift from one provider to the next. This aligns with global recommendations advocating for adolescent-centred transition models incorporating ongoing support, monitoring and flexible service adaptations throughout the HIV care continuum.^10,11^ Thus, the transition to adult HIV care cannot be solely addressed at the programmatic level but requires system-wide interventions addressing resource constraints, service fragmentation and weak referral systems.^42,43,46^

### Strengths and limitations

A key strength of this study is its focus on healthcare worker perspectives from diverse clinical backgrounds across the Cape Town Metropole, offering rich insights into transition practices across settings. This diversity of frontline insight further offers how practices are implemented in real-world clinical settings. This study further highlights the variability of service responses and identifies system-level barriers that are often underreported in existing literature. The limitations of this study include the homogeneity of the participants and its geographic focus, which may limit the generalisability of this study. Future research should examine practices of providing care to adolescents living with HIV in the transition process among rural and underrepresented provinces to deepen understandings of transition support across the healthcare system in South Africa.

### Implications for future research and recommendations

This study highlights critical opportunities to strengthen transition practices for adolescents living with HIV by addressing individual, systemic, service delivery and programmatic gaps. Healthcare workers emphasised the need to implement standardised transition protocols and readiness assessments, supported by early disclosure and structured psychosocial interventions. The formalisation and scale-up of peer-led programmes, which include youth clubs and peer mentorship, are essential for sustaining engagement across the transition and HIV care continuum. When integrated into both paediatric and adult HIV services, adolescent-centred approaches can significantly improve adherence and retention. The findings also underscore the urgency of formalising national and provincial policy guidelines on transition and the investment in capacity-building of adult HIV services to meet the specific needs of adolescents living with HIV. Future research should evaluate the effectiveness, scalability and sustainability of context-specific transition practices and interventions, particularly in under-resourced settings. Implementation science-driven research is needed to explore how adolescent-centred transition models can be integrated into routine health system processes and supported within resource constraints.

## Conclusion

Transitioning adolescents living with HIV to adult care is a complex, multifaceted process shaped by individual readiness, psychosocial dynamics and structural health system factors. This study underscores the need for coordinated, adolescent-centred services extending across all stages of the transition process. Key impediments this study identified include delayed disclosure, limited transition preparation and the absence of formalised protocols, all of which jeopardise engagement in care and health outcomes. Despite these challenges, healthcare workers highlighted several enablers of successful transitions. This includes strong provider–adolescent relationships, peer support and youth-friendly service environments. However, these are inconsistently implemented and often reliant on individual effort rather than systemic support. A sustained health systems response grounded in policy, training and adolescent engagement is essential to ensure that adolescents living with HIV are not only engaged in care but also are supported to thrive as they emerge as optimal, economically contributing adults.
